# Illuminating HBV with multi-scale modeling

**DOI:** 10.3389/fsysb.2023.1045754

**Published:** 2023-02-20

**Authors:** Shawn A. Means, Md A. Ali, Harvey Ho

**Affiliations:** ^1^ Auckland Bioengineering Institute, University of Auckland, Auckland, New Zealand; ^2^ Department Mathematics and Physics, Kansas Wesleyan University, Salina, KS, United States

**Keywords:** liver, sinusoid, HBV, zonation, spatial, multiscale, mathematical model HBV hepatatis-B virus

## Abstract

Unfortunately for the estimated 250 million sufferers of chronic hepatitis-B viral (HBV) infection worldwide, the liver terrain is typically ignored. An immuno-tolerant environment attractive for pathogens, the essential metabolic roles and structural features of the liver are aligned with distinctive gradients of oxygen and nutrients established along blood flows through fundamental hepatic processing units known as sinusoids. Capillaries surrounded by banks of hepatocytes, sinusoids express spatial configurations and concentrations of not only metabolic roles but also immune cell localisations, blood filtering and transporter specialisations: the liver terrain. HBV targets proteins regulating gluconeogenesis, a crucial liver function of blood glucose management, highly active at blood entry points—the periportal sites of sinusoids. Meanwhile, at these same sites, specialised liver macrophages, Kupffer cells (KC), aggregate and perform critical pathogen capture, detection and signaling for modulating immune responses. In tandem with KC, liver sinusoidal endothelial cells (LSECs) complement KC blood filtration and capture of pathogens as well as determine KC aggregation at the periportal sites. Failure of these systems to establish critical spatial configurations could ironically facilitate HBV invasion and entrenchment. Investigating the impacts of spatial and structural variations on the HBV infection dynamic is experimentally challenging at best. Alternatively, mathematical modeling methods provide exquisite control over said variations, permitting teasing out the subtle and competing dynamics at play within the liver terrain. Coordinating with experimental observations, multi-scale modeling methods hold promise to illuminate HBV reliance on features of the liver terrain, and potentially how it may be defeated.

## 1 Introduction

Chronic hepatitis B viral infection (HBV) afflicts some 250 million people in the world today (Schweitzer et al., 2015). Eliminating the virus from liver tissue altogether is nearly impossible partly due to the notorious tenacity of HBV in establishing persistent infection, and treatment options aimed at controlling the disease are quite limited. Chronic infection typically arises because of perinatal transmission, but also the often-successful clearance of ‘acute’ HBV infection can lead to chronic in a small proportion (roughly 5%) of instances (Rehermann, 2013). Chronic HBV (CHB) is marked by establishment of the viral DNA in host hepatocyte nuclei with remarkably stable, covalently closed circular DNA (cccDNA). Levels of persistent cccDNA may remain undetectably low in CHB sufferers (Schmeltzer and Sherman, 2010), leading to disease resurgence when the viral-immune balance is disrupted with, for instance, chemotherapy immunosuppression (Hoofnagle, 2009). Tissue damage of fibrotic and cirrhotic pathologies along with carcinomas of the liver can further result (Ikeda et al., 2009), further complicated by, for instance, myriad influences of alcohol on HBV (Muro et al., 2022). Overall, approaches for treatment are aimed at suppression of the virus, such as reverse transcriptase inhibitors, requiring enduring long-term protocols that fail to eradicate cccDNA –leading to the resurgence of HBV once treatment ends. Transplantation or transfusion aimed at eliminating HBV may further fail due to the cccDNA levels (some under 100 copies/mL) falling below detection, leading to resurgent HBV activity with corresponding complications for post transplantation rejection (Minguela et al., 2006).

The mystery of why 95% of acute infections resolve yet the remaining 5% manifest in CHB is rooted in our poor understanding of the immune response to HBV. Both the innate and adaptive immune responses interacting with the virus are essential to the final infection outcome, but are both suspected responsible for facilitating HBV in various ways. First-line innate defences of liver-resident macrophages, the Kupffer cells (KC), critically filter and capture myriad pathogens as they flow through the gut-liver axis, and are suggested central to HBV ‘stealth’ infection when they permit viral entry (Chang and Guo, 2015). Failure to capture HBV upon entry to liver sites may be connected to their unique and spatially-localised role: if out of position, KC may permit pathogens to opportunistically colonise hepatocytes (Gola et al., 2021). Alternatively, successful guardianship of the liver by the innate response must be complemented by the adaptive response such as with CD4+/CD8+ T-Cells. Specific targeting of HBV-infected hepatocytes is crucial to the clearance of the virus into an acute transient infection—barring any excessive cytolytic damage (Chisari et al., 2010).

Complicating the immune response picture are modulations between the innate and adaptive that emerge as counterproductive or even internecine. Innate cells such as the Natural Killer (NK) surprisingly may eliminate CD8^+^ T-cells when triggered to release interferons (Guidotti et al., 2015), or outright block apoptotic mechanisms of infected cells greatly frustrating efforts at viral clearance (Hösel et al., 2009). The complex milieu of cytokines enabling communications between the innate and adaptive immune branches as well as parenchymal cells in the liver may either facilitate or frustrate HBV infection. Resultant levels of inflammation corresponding to levels of interferon (IFN-gamma) along with extensive activation of immune responses in the liver show a remarkable correlation to CHB (Johnson Valiente et al., 2022). Acute infections, meanwhile, do not demonstrate such an extensive immune response, suggesting overzealous immune activation is the culprit, or at least a conspirator, as it were.

**FIGURE 1:**
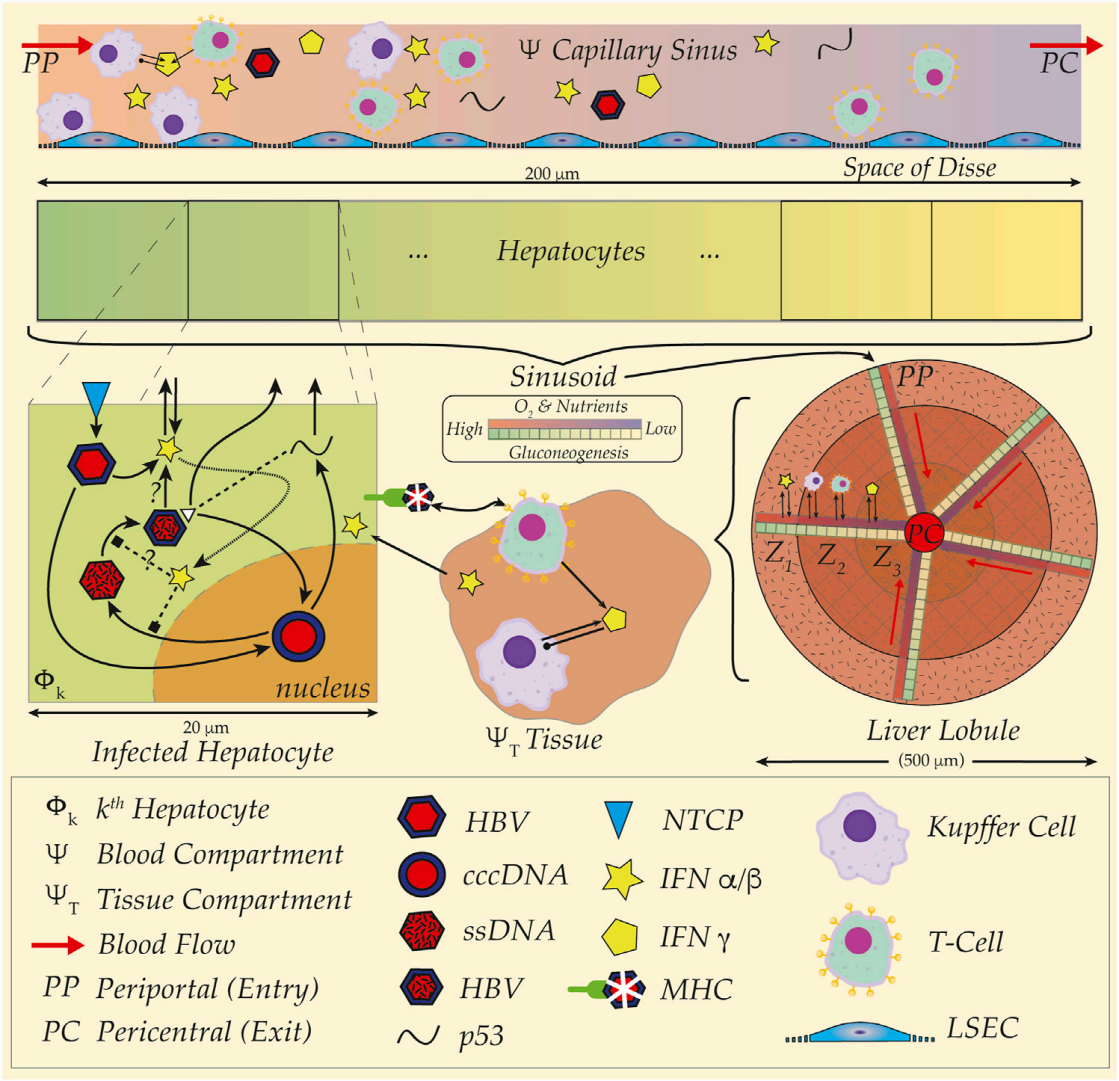
HBV and spatial organisation, distribution and structure of liver sinusoid. Cartoon schematic illustration of multiple spatial scales and organisation of liver from hepatocyte (20 um) up to sinusoid (200 um) and liver lobule (500 um). (Top Rows) Gradients of O_2_ and nutrients represented in capillary sinus blood (*Ψ*) with colour gradient (red to blue) from Periportal (PP) to Pericentral (PC) site. Metabolic activity illustrated with colour gradient (green to yellow) for gluconeogenesis in hepatocytes. Note blood sinus populated with Kupffer Cells (purple), T-cells (green) as well as full HBV virus (red and blue hexagons), and representations of the immune cytokines interferon alpha/beta (IFN α/β, yellow star) and interferon gamma (IFN γ, yellow pentagon). Kupffer cells (KC) aggregate at the PP site for detection and capture of pathogens, further modulating downstream immune cell responses. Acinar assemblies of hepatocytes interface with materials transported to the Space of Disse by way of fenestrated liver sinusoidal epithelial cells (LSECs, low-profile blue cells in sinus)—either passively filtered or actively bound and uptaken from proximal and slow blood flows at around 100 um/second. An example HBV infection dynamic is illustrated (Mid-Left, ‘Infected Hepatocyte’), where HBV invades hepatocyte *via* the NTCP transporter (blue diamond) and establishes cccDNA in the nucleus (red-blue circle). Viral proteins such as p53 and precursor viral DNA (ssDNA, mottled red hexagon) later assembled into full HBV virion and released back to sinus. Intracellular interactions with interferons illustrated with unknown potential modes of action, as well as presentation of viral sequences with the major-histocompatibility complex (MHC) to surveilling immune cells in the surrounding hepatic tissue (Region *Ψ*_T_). Interferons and interleukins establish communications and source of coupling between compartments encompassing immune and endothelial cells either in tissue or in blood. Sinusoidal organisation of metabolic labour and immune cell aggregations arrayed according to said gradients within sinusoid including liver lobule zonation (Z_1_, Z_2_, Z_3_); note radial arrangements of capillary sinuses emptying to the pericentral site with corresponding hepatocyte arrays (Liver Lobule, Mid-right). Outermost tissue region, Z_1_, with richest blood with corresponding highest gluconeogenesis and innermost, Z_3_, with most depleted, corresponding to particular metabolic activity, such as paracetemol processing. Individual transcription modulators such as the protein PGC1-alpha hijacked by HBV for viral DNA replication at higher levels of activity in PP region corresponding to elevated gluconeogenesis in the same region (see text).

Experimentation aimed at teasing out the dynamics of these systems is challenged not only by the complications of immune responses, but the influence of the hepatic microenvironment. Extricated from the liver, cultured cells cease to display characteristics endemic to their functional roles; for instance, Cyp450 enzyme activity disappears mere days after culture, whereas phases of HBV activity span over weeks (Zeisberg et al., 2006; Chen et al., 2012; Rowe et al., 2013). Microenvironments in the liver—fluid flow down capillary blood systems surrounded by banks of hepatic cells, or sinusoids (see Figure 1)—establishing gradients of O_2_ and nutrients exert a profound influence on hepatocyte morphology and behaviour. Assemblies reproducing hepatic 3D microfluidic environments maintain cellular differentiation for over a month with demonstrable innate immune activations upon HBV inoculation (Ortega-Prieto et al., 2018). Nevertheless, such a remarkable experimental apparatus still isolates a faux liver environment from a full *in vivo* response to HBV challenge triggered by extra-hepatic systems such as in the spleen or lymph node (Yoffe et al., 1990; Mason et al., 1993; Gao et al., 2017). Combined with difficulty in obtaining human patient data—particularly excised tissue—and restrictions on primate animal models, mathematical and *in silico* studies present alternative approaches enabling exquisite control over details such as structural and spatial dimensions simply unavailable to the experimentalist.

## 2 A spatial frontier: Liver structure and HBV

HBV invasion and confrontation by immune responses occur in a remarkably complex hepatic landscape known for its spatial organisation. In 1992, Gebhardt described metabolic roles arranged according to proximity with blood flows against the background of hepatic lobe structures (Gebhardt, 1992). Enriched with oxygen and nutrients at portal inlets, blood enters hepatic lobes and encounters narrow capillaries confining flow rates to below 100 um/s (Wong et al., 1997), while filtration, extraction and metabolic activity successively deplete the enriched blood before exit at the pericentral outlet (Figure 1). Gradients formed by these operations establish ‘zones’ in hepatic lobes with respectively higher levels of O_2_ and nutrients near inlets and hypoxic and nutrient-deficient regions in the lobe interior. Recent observations confirm the zonal description permeates down to cellular metabolic activity and a division of labour across these liver landscapes (Gebhardt and Matz-Soja, 2014; Halpern et al., 2017) including the application of spatial proteogenomics and transcriptomics to not only the metabolic activity, but also the immune (Hildebrandt et al., 2021; Guilliams et al., 2022).

Resident hepatic macrophages—the KC—preferentially aggregate around the portal inlets (Baratta et al., 2009; Gola et al., 2021; Guilliams et al., 2022); a tactically advantageous position for their role in filtration and capture of pathogens, shielding the downstream hepatocytes from infection. Situated thus, KC act as watchmen releasing cytokines such as interleukin-6 (IL-6) and tumour necrosis factor (TNF) upon encounters with HBV (Hösel et al., 2009; Broering et al., 2021) apparently key to successful innate immune response. Failing to station at these critical entry points, KC are suggested far less effective at their defensive roles, where pathogens successfully colonise deeper in the sinusoidal array and beyond (Gola et al., 2021). Moreover, if out of position, KC may further disrupt the crucial balance of cytokine signaling responses to HBV challenge: failure of HBV detection may lead to suppression of immune response by KC release of interleukin-10 (IL-10) (Liu et al., 2018; Broering et al., 2021). Ironically, the ‘watchmen’ of the sinusoid may thus signal all is well while viral invaders breach the hepatic walls—facilitating HBV establishment.

Of particular note is the mechanism establishing KC aggregation in the sinusoid at the periportal site. Complementary ligands and receptors between endothelial cells lining the sinusoid, the Liver Sinusoidal Endothelial Cell (LSEC), and KC emerged in a study by Halpern, et al. (Halpern et al., 2018). Evidently, KC preferentially adhere at the LSEC regions where suitable complements of ligands and receptors express, as suggested later by Gola, et al. (Gola et al., 2021).

Sitting between the sinusoidal lumen and banks of hepatocytes, LSECs perform mundane yet critical tasks of uptake, filtration and transport for metabolisation (See Figure 1). Fenestrated orifices between blood and the space of Disse as well as endocytotic mechanisms facilitate LSEC selection and scavenging of molecules for presentation to hepatocytic surfaces [see, for instance, Sorensen, et al. (Sørensen et al., 2012)]. LSECs further perform roles analogous to those in lymphocytes expressing similar surface molecules while aiding lymphatic drainage within the Space of Disse (Knolle and Wohlleber, 2016). Not only do LSECs encourage adherence of KCs in specific regions, they also work in tandem with immune surveillance by capturing passing pathogens with receptors analogous to those on KCs probing the proximal blood flows (Ander et al., 2022). Mannose receptors on LSECs (LSECtin) for uptake of molecules comprising mannose residues, also bind T-cell surface molecules (CD44) and facilitate T-cell activation; however, LSEC-T-cell interfaces may also override dendritic cell activation of T-Cells (Knolle and Wohlleber, 2016). LSECs are yet another player in the liver immune surveillance and response dynamic involved in the modulation of spatial localisation, pathogen detection and immune cell activation.

Continuing the spatial organisation theme, LSECs activities arrange according to the established gradients of O_2_ down the sinusoid. Observations report high production of lactate by LSECs (Martinez et al., 2008) suggesting a preferential metabolic activity in hypoxic regions—such as the pericentral zone. Alternatively, LSECs exposed to higher O_2_ levels as found in the periportal express enhanced production of IL-10 (Sørensen et al., 2012) apparently in coordination with proximal KC for immune downregulation. Chemokine gradients are further connected with LSECs and their apparently varying roles down the sinusoid (Gola et al., 2021).

Corresponding with the preferential arrangements of KC at the periportal inlet, the action of gluconeogenesis—the essential metabolic regulation of blood glucose—operates at peak levels (see Figure 1) (Gebhardt, 1992; Halpern et al., 2017) and is targeted by HBV. Specifically, the modulator of genes for gluconeogenesis, the protein PGC1-alpha, is manipulated by HBV for viral transcription (Bar-Yishay et al., 2011). PGC1-alpha sits at the hub of a protein and gene network comprising essential transcriptors and co-activators fundamental to hepatocyte and liver metabolic nutritional maintenance. Defective PGC1-alpha is linked with numerous nutritional abnormalities and morbidities (Leone et al., 2005), and subjected to HBV exploitation for viral replication (Shlomai et al., 2006; Jhuang et al., 2015; Shalaby et al., 2017) suggested the interactions between the virus and this key metabolic modulator may be instrumental to HBV-induced hepatic steatosis (Bar-Yishay et al., 2011). Against the backdrop of KC localisations at the same peak levels for gluconeogenesis—and inferentially PGC1-alpha activity—successful clearance of HBV may critically hinge on KC correctly situated for detection and immune response activation in tandem with LSEC filtration and capture of HBV before passage to the space of Disse.

HBV further exploits the sodium-taurocholate co-transporting polypeptide or NTCP for cellular invasion (Watashi et al., 2014), and this transporter may be spatially arranged in correspondence with metabolic roles across the lobular zones. Measurements of mRNA sequencing at distinct lobule locations demonstrated the potential of non-randomly zonated gene expressions (Halpern et al., 2017). Some of the SLC genes studied exhibited a heterogeneous distribution down the sinusoidal axis with higher expressions at the periportal than in the pericentral.

Co-alignment of several spatially organised actors and components including: *i*) immune cell surveillance and signaling in key spatial locales; *ii*) LSEC guidance of immune cell localisation, complementary blood filtration and signaling; and *iii*) distribution of metabolic roles down the sinusoid with localised exploitation by HBV, all demonstrate an intrinsic spatial character of HBV infection dynamics. This spatial character has largely gone unexplored, yet holds a tantalising possible solution to the CHB *versus* acute clearance conundrum: failure of LSECs to successfully capture KC in necessary locations for essential surveillance may be fundamental to disruption of immune cell signaling. Potentially, such failure may even ironically facilitate HBV entrenchment by virtue of KC immune response suppression.

## 3 Mathematically modeling the spatial possibilities

Establishing such possible scenarios experimentally is not trivial; these complex and complementary spatial configurations present significant challenges for their investigation. Particularly, varying said configurations and observations of their impacts that are difficult if not impossible in experimental contexts are, by contrast, quite straightforward with mathematical and computational *in silico* methods. A mathematical model can readily assign KC concentrations and distributions to different regions down the sinusoid as well as LSEC activities of filtration or capture and interactions with the hepatocytes downstream of transport. The hepatocyte metabolic activity—and by the same token HBV replication rates—can also be varied in suitable spatial gradients with peaks at the periportal, or the reverse if so desired. Typically, however, these spatial characteristics and their profound influence on HBV infection and immune responses are mostly ignored in mathematical models of HBV (Means et al., 2020).

Non-spatial mathematical models are not without their utility, however. Based on ordinary differential equations (ODEs), well-mixed mathematical models such as the classic Nowak, et al. treat the liver as a spatially homogenous compartment including uninfected and infected hepatocytes as well as active virus in dynamically coupled populations (Nowak et al., 1996). As noted in the Ciupe review of HBV models, ODE models were instrumental in characterising multiple HBV aspects such as virus production, clearance, and infected hepatocyte life-spans (Ciupe, 2018). The relative ease and wide array of tools available for ODE simulation and analysis prompt their application to HBV dynamics and are thus a preferred approach for systems avoiding undue complexity (Ciupe and Heffernan, 2017). Nevertheless, ODEs are necessarily limited to systems with no spatial variation, and the liver is a profoundly spatially organised system as described above.

Little attention to the spatial component of HBV dynamics is found in the mathematical modeling literature. A sequence of models analysed the addition of spatial diffusive transport for HBV in tissue showing a minimal impact on the stability of underlying ODE models (Wang and Wang, 2007; Wang et al., 2008; Xu and Ma, 2009). Later additions of diffusion for antibody, virus and virus + antibody complexes by Huang, et al. (Huang et al., 2019) analysed the resulting system of ODEs and partial differential equations (PDEs) but did not conclusively determine the impact of diffusion on infection persistence, or the reproduction number, *R*_*0*_. None of these diffusion transport models address either advective blood transport or the crucial and distinctly spatially organised liver metabolic architecture. Meanwhile, a comparable viral infection dynamic of influenza in the respiratory tract attracted an effort including effects of both advective and diffusive transport by Quirouette et al. (Quirouette et al., 2020). They showed that the application of non-spatial mathematical models to the viral dynamic likely underestimates the key viral production rate with corresponding errors for total virus produced over the course of infection as well as emergence of any drug resistances. Well-mixed ODE models may be relatively accessible for analysis and computation, but they may also be quite insufficient when spatial elements are influential as in the liver.

Yet, pharmacological processing against the backdrop of liver structure and critical aspects of intrinsic spatial configurations in sinusoids has not gone unnoticed. These inspired proposals of spatial modeling frameworks for precisely these issues by Schwen, et al. (Schwen et al., 2015) and Sluka et al. (Sluka et al., 2016), or the cellular automata schemes as presented by Adhyapok et al. (Adhyapok et al., 2020). Drug perfusions illustrate the subtle yet profound influence of the liver spatial landscape on, for instance, hepatic steatosis during initial treatments (Schwen et al., 2015). Further, specific regions of toxicity for common analgesics such as paracetamol emerge due to spatially-organised metabolic labour: pericentral regions display concentrated damage when exposed to overdose (Umbaugh et al., 2021) as predicted by a spatial mathematical model (Means and Ho, 2019) and the cellular automata (Adhyapok et al., 2020).

The frameworks proposed by Schwen, et al. and Sluka, et al. construct sinusoidal regions sampling whole organ activity rather than attempting to capture the entire liver action. Blood flows and transport of material are represented with capillaries in sinusoidal spaces surrounded by banks of hepatocyes exposed to these materials with interactions shaped by concentrations and metabolic roles distributed according to spatial locale. Equations for the mathematics are one-dimensional advection diffusion for blood transport coupled with systems of ordinary differential equations installed in individual hepatocytes for metabolism. Given sinusoidal length of ∼200 um, a relatively small population of roughly 30 cells are typically arrayed in the scheme.

Expansion of the sinusoidal framework beyond the continuum differential equations and their limitations to average concentrations is necessary for tracking the notoriously low levels of HBV particles often falling below detection in occult cases (Guo and Guo, 2015). Hence, agent-based methods for computing distributions of HBV particle counts in individual hepatocytes such as presented by Murray and Goyal (Murray and Goyal, 2015) were adapted into the sinusoidal framework for our spatial HBV model (Cangelosi et al., 2017). Investigating the impact of varying spatial distributions for transport, replication and immune-system responses in sinusoids illustrated the importance of suitable—and spatially-localised—immune activity for clearance of HBV (Cangelosi et al., 2017). Without appropriate spatial responses, HBV escapes clearance and establishes chronic infections—as suggested in the above discussion. Nevertheless, our spatial model for HBV-immune dynamics included significant limitations, particularly with highly idealised immune responses. The complex interplay between KCs, LSECs and hepatocytes as well as cytokine communications call for additional creative application of methods such as agent-based or cellular automata for individual cells such as KC-LSEC aggregation mechanisms.

LSECs themselves prompt consideration of additional layers for cells in the sinusoidal framework (Figure 1). As ‘man-in-the-middle’ actors, filtering transport of blood-borne particles for presentation to hepatocytes, combined with spatial organisation of LSECs themselves down the sinusoid calls for their inclusion. Impact of LSECs on dynamics of metabolism or infection appear considerable as well as LSEC roles in the capture and degradation of circulating pathogens. Crosstalk and interaction with KCs and downstream cytokine signaling and LSEC influence on KC spatial positioning appear key to pathogen detection.

## 4 Discussion

Although a daunting overall challenge, the essential and influential spatial organisation of hepatic activity calls for the assembly of multi-scale mathematical models of the sinusoid. From intracellular viral infection and replication interacting with interferons, to KC detection or failure thereof and downstream cytokine signaling, to transport in the blood throughout the tissue and body with extra-hepatic exposures then back again, decrypting HBV infection activity is an intrinsically multi-scale dynamic. Tractability is possible for computing model solutions by using sampling sinusoidal models scattered over an idealised, *in silico* liver organ. Continuum methods for tracking concentrations of cytokines combined with agent-based methods for viral particle influences as well as individual cell type behaviour can feasibly operate against the background of spatially distributed components. Metabolic rates, viral infectivity and replication that differ over the sinusoid with concentrated or diffuse immune responses in specific locations appear instrumental for successful liver clearance of pathogens. These levels of models can be dropped into a PBPK-type formulation with extrahepatic infections or replications in the spleen or other organs exposed to the virus and responses with cytokines traversing back to the liver. Further investigating the hepatic domain’s crucial spatial and structural components necessarily requires some manner of testing impact of their variants. Mathematical multi-scale methods enable precisely this approach—when suitably and carefully constructed for computational efficiency.

Nevertheless, no mathematical model prediction is useful unless anchored in actual experimental data. Although animal models and *in vitro* experiments cannot approach the level of precision for such a multi-scale mathematical scheme, parameters for infection dynamics, transport and activity must necessarily derive from measurements or at least sit in realistic ranges. Sensitivity analysis determining the dominant influence of parameters within the framework of these multi-scale mathematical models can inform fruitful directions for experimental focus (Ali et al., 2021). Fortunately, extraordinary advances in experimental work with spatial proteogenomics and transcriptomics are encouraging; detecting previously unavailable distributions for numerous hepatic activities can naturally inform the multi-scale model described here. Based on such observations, the multi-scale model can then precisely vary observed distributions and inspect their varied influences. Coordinating efforts between the experimental and theoretical branches, we may finally illuminate the precise mechanisms leading to establishment of CHB in hundreds of millions of sufferers across the globe.

## Data Availability

The original contributions presented in the study are included in the article/supplementary material, further inquiries can be directed to the corresponding author.

## Abbreviations

HBV, Hepatatis-B Virus; CHB, Chronic HBV; cccDNA, Closed circular DNA; KC, Kupffer Cells; NK, Natural Killer; TNF, Tumour Necrosis Factor; IL-6, Interleukin 6; IL-10, Interleukin 10; LSEC, Liver Sinusoidal Endothelial Cells; NTCP, Sodium-Taurocholate Co-transporting Polypeptide; PBPK, Physiologically-based Pharmaco-kinetic.
